# Protocol for the use of focused ion-beam milling to prepare crystalline lamellae for microcrystal electron diffraction (MicroED)

**DOI:** 10.1016/j.xpro.2021.100686

**Published:** 2021-07-28

**Authors:** Michael W. Martynowycz, Tamir Gonen

**Affiliations:** 1 Howard Hughes Medical Institute, University of California Los Angeles, Los Angeles, CA 90095, USA; 2 Department of Biological Chemistry, University of California Los Angeles, Los Angeles, CA 90095, USA; 3 Department of Physiology, University of California Los Angeles, Los Angeles, CA 90095, USA

**Keywords:** Microscopy, Protein Biochemistry, Structural Biology, Cryo-EM

## Abstract

We present an in-depth protocol to reproducibly prepare crystalline lamellae from protein crystals for subsequent microcrystal electron diffraction (MicroED) experiments. This protocol covers typical soluble proteins and membrane proteins embedded in dense media. Following these steps will allow the user to prepare crystalline lamellae for protein structure determination by MicroED.

For complete details on the use and execution of this protocol, please refer to Martynowycz et al. (2019a, 2020a).

## Highlights

• Protocol for focused ion-beam milling of protein crystals for MicroED experiments
• Detailed steps to generate crystalline lamellae
• Procedures for MicroED data collection
• Optimization and troubleshooting for highest resolution possible

## Graphical abstract

**Figure:**
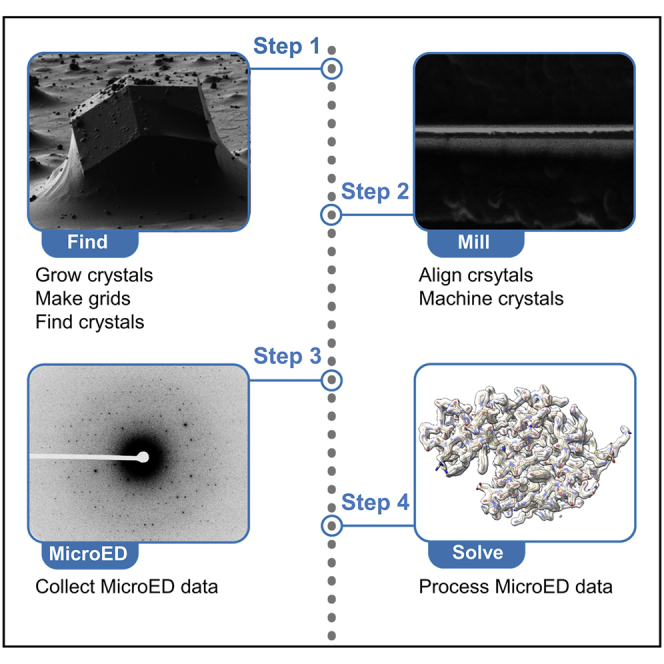

## Before you begin

Focused ion-beam milling is used to generate ideal specimens for MicroED experiments. Preparing lamellae from crystals is advantageous to standard sample preparation methods (de la Cruz et al., 2017; Shi et al., 2016), since it removes excess material that contributes to noise, makes locating prepared samples easy in subsequent steps, and allows for some control of sample geometry prior to collecting MicroED data. General instructions on the operation of the TEM and dual-beam instruments to prepare lamellae of non-crystalline materials are described elsewhere (Marko et al., 2007; Medeiros et al., 2018; Wagner et al., 2020). The general workflows and approaches of milling samples prior to collecting cryo-tomography data are overall similar (Buckley et al., 2020; Gorelick et al., 2019; Zachs et al., 2020), but there are differences when preparing crystalline lamellae (Martynowycz et al., 2020a). Focused ion-beam milling is a general approach to sample preparation for MicroED samples (Beale et al., 2020; Duyvesteyn et al., 2018; Li et al., 2018; Martynowycz et al., 2019a, 2019b; Polovinkin et al., 2020; Zhou et al., 2019). Before starting, it is required to have protein crystals or microcrystals grown and ready for experiments. Generation of standard crystals of tetragonal hen egg white lysozyme are presented as an example and for instructional purposes. It is assumed that the dual beam FIB/SEM for milling the crystals, the TEM for MicroED data collection are well-aligned and operating at cryogenic temperatures, and that the user is familiar with vitrification robots and basic cryo-EM sample grid preparation. Further reading on operation of vitrification robots and procedures are described elsewhere (Carragher et al., 2019; Grassucci et al., 2007; Passmore and Russo, 2016).

## Key resources table

**Table:** 

REAGENT or RESOURCE	SOURCE	IDENTIFIER
**Chemicals, peptides, and recombinant proteins**		

Sodium acetate 1M pH 4.7	Hampton Research	CAT NO: HR2-233
5M Sodium chloride stock solution	Hampton Research	CAT NO: HR2-637
Lysozyme	Millipore Sigma	L4919-500MG CAS: 12650-88-3

**Deposited data**		

Atomic coordinates of lysozyme determined from fragmented protein crystals using MicroED	de la Cruz et al. 2017	PDB: 5K7O
Atomic coordinates and structure factors of lysozyme determined by MicroED from milled lamellae	This paper	PDB: 7MRP
Density maps of lysozyme determined by MicroED from milled lamellae	This paper	EMDB: EMD-23957

**Software and algorithms**		

MAPS	Thermo Fisher Scientific	N/A
EPU-D	Thermo Fisher Scientific	N/A
MicroED tools	cryoem.ucla.edu	RRID:SCR_021179
XDS	xds.mr.mpg.de	RRID:SCR_015652
PHASER	www.phaser.cimr.cam.ac.uk	RRID:SCR_014219
COOT	https://www2.mrc-lmb.cam.ac.uk/personal/pemsley/coot/	RRID:SCR_014222
PHENIX.REFINE	phenix-online.org	RRID:SCR_014224

**Other**		

TEM grids	Quantifoil	Cu200 R2/2
Glass cover slides	Ted Pella	Prod# 26003
Syringe filters	Sigma-Aldrich/Whatman Puradisc 0.45μm	SKU WHA10462600
Various forceps	Ted Pella	N/A
Parafilm	Parafilm	N/A
1, 10, 200, 1000 μL pipette and disposable tips	Eppendorf	N/A
Transmission electron microscope	Thermo Fisher Talos Arctica	RRID:SCR_019905
Dual-beam focused ion-beam and scanning electron microscope (FIB/SEM)	Thermo Fisher Scientfic	RRID:SCR_019880
Vitrification robot	Leica EM GP2 (used here) Thermo Fisher Vitrobot Manual plunger	N/A N/A N/A
Glow discharger	Pelco	RRID:SCR_020396

## Step-by-step method details

In this protocol we describe all the steps of determining protein structures of ion-beam milled crystals by microcrystal electron diffraction (MicroED).

### Preparing protein crystals

**Timing: 1–2 h to prepare; 1–10 days for crystals to grow**

This step describes generating macromolecular protein crystals of lysozyme required for ion-beam milling and subsequent MicroED investigation. We include these steps and instructions to follow if this protocol is being used as a learning resource. Lysozyme is used as a standard test sample, and it is otherwise expected that these steps may be skipped if protein crystals are already grown and ready for experiments.

1. Prepare the protein stock solution
  a. Dissolve dry powder of lysozyme into a buffer of 0.1 M sodium acetate pH 4.8 solution to a concentration of 40 mg / mL.
  b. Filter this protein solution through a 0.4 μm syringe filter at least 3 times
  c. Aliquot the protein into 50 μL portions in PCR tubes and flash freeze in liquid nitrogen
  d. Store at −80°C until just prior to use
    i. Thaw the protein on ice prior to experiments, and spin the down the aliquots at 1–5,000 RPM on a tabletop centrifuge to remove aggregates
2. Prepare the precipitant solution
  a. Create a stock solution of 4 M NaCl using filtered water
    i. Filter the salt solution through a porous membrane at least 3 times
  b. Create a stock solution of 1 M sodium acetate pH 4.8 using filtered water
    i. Similarly filter to assure purity and remove aggregates
  c. Mix 1 mL of sodium acetate with 2.5 mL NaCl stock and 6.5 mL filtered water to create a 1 M NaCl 0.1 M sodium acetate pH 4.8 precipitant solution
3. Crystallize the protein
  a. Mix 1–5 μL of protein stock solution with precipitant solution in a 1:1 ratio inside of a PCR tube
  b. Gently mix with a pipette for 30 s
    i. Crystals form in 1–10 days at approximately 23°C

***Note:*** Batch crystallization by mixing solutions can be replaced by sitting or hanging drop vapor diffusion in crystallization plates, and is typically faster. Batch conditions can simplify the downstream sample preparation processes.

**Pause point:** days

### Preparing TEM grids for cryo-FIB milling

**Timing: 0.5–2 h**

These steps describe precisely how to prepare TEM grids. It involves applying the protein crystals to a glow discharged grid, blotting away the excess solvent, and plunging the crystals into liquid ethane.

4. Wrap one side of a glass cover slide with parafilm wrap and place the cover slide gently into a glass petri dish lined with dry filter paper
5. Open a container of TEM grids and gently remove them one at a time by their edge, placing them onto the parafilm wrapped side of the cover slip with their carbon side facing upward (Figure 1)
  a. TEM grids typically have a carbon side and a copper side. The copper side is reflective and bright, whereas the carbon side is darker and appears more drab. Typical TEM grid containers have the grids oriented with their carbon sides facing the center of the package.

**Figure 1:**
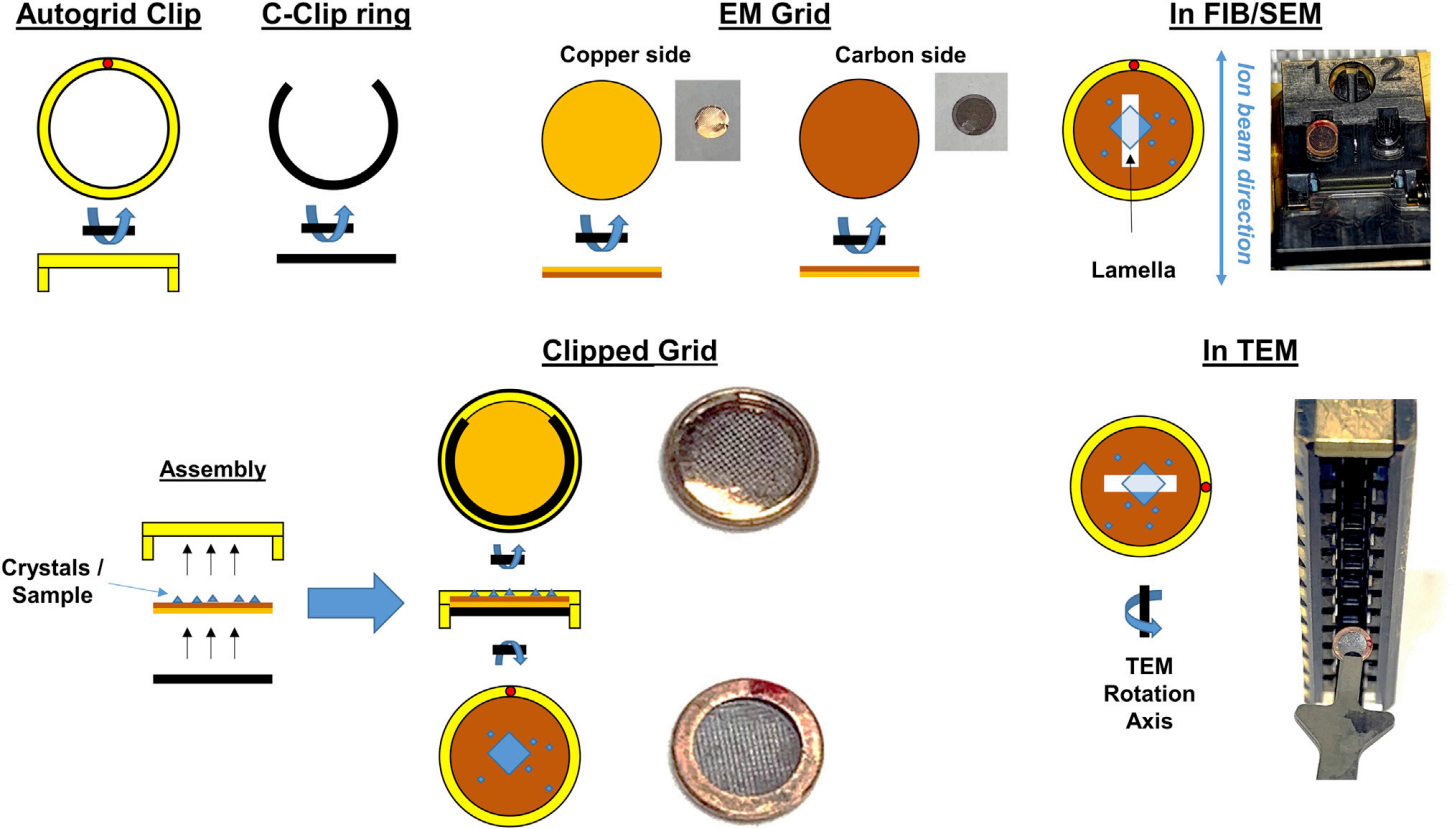
EM Grids and how to assemble them for milling crystals Schematic cartoons of the grids, assembly, and preferred orientation of the clipped EM grids are presented along with images for reference.
6. Gently place the cover slide with the TEM grids into a glow discharge chamber and replace the cover to assure a vacuum-tight seal
7. Glow discharge the grids on the negative setting for 10–30 s at a currant of 15 mA
8. Place the cover slide back into the glass petri dish. Make sure to replace the glass cover to prevent dust or contamination from building up on the grids.
9. Use the mountable forceps to grab a TEM grid by the outer edge, assuring not to puncture the inner portion with the tips of the forceps. Lock the forceps closed on the grid and place it into the vitrification robot.
10. Move the forceps into the sample chamber of the vitrification robot and allow the grid to equilibrate with the chamber environment for approximately 1 min.
11. Using a 10 μL pipette, place approximately 1–3 μL of solution containing crystals onto the carbon side of the TEM grid inside of the vitrification robot sample chamber
12. Wait 20 s and then blot the TEM grid gently from the back for approximately 20 s
  a. 20 s is a typical blot time when the robot is at 4°C and 95% humidity. Optimization of this parameter and the environment inside the vitrification robot chamber are often required.
13. Plunge the blotted TEM grid into super-cooled liquid ethane to vitrify the liquid and crystals on the grid
14. Transfer the grid to a grid storage box
15. Repeat steps 6–11 to prepare as many samples as needed
  a. It is often useful to make at least 4 samples at a time, as this is how many grids fit into a single grid box, and allows for trying several variations of grid preparation at a time – e.g., blotting for 4 s, 8 s, 16 s, 20 s, etc.
16. Transfer the grid boxes into a liquid nitrogen storage dewar prior to use

**Pause point:** Months

### Cryo-FIB/SEM investigation of protein microcrystals

**Timing: 2–12 h at a time**

These steps detail the method of screening grids for microcrystals in the SEM and FIB beam, aligning selected samples, and milling protein crystals into thin lamellae for subsequent MicroED investigation.

**Figure 2:**
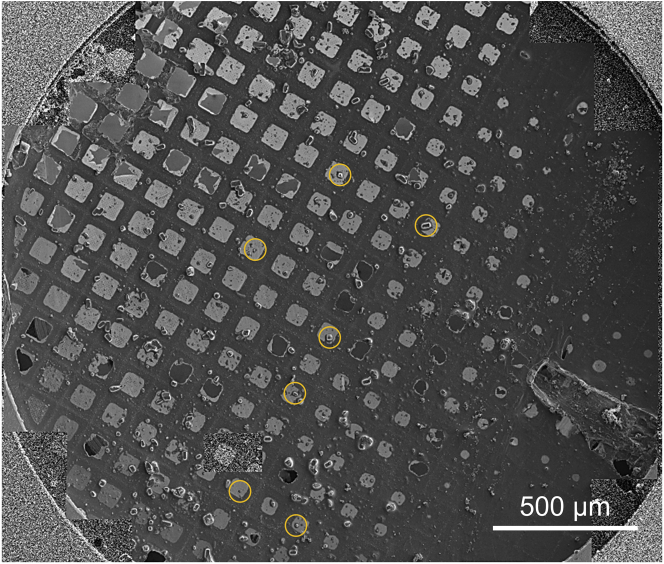
Overview of an EM grid by using the SEM A montaged of individual SEM images stitched together in order to inspect the grid for potential crystals. The seven crystals identified for this investigation are circled in orange.
  b. Identify crystals that are not on top of grid bars, not within 3 grid squares of the edge of the sample, and that are on unbroken/bend/ripped grid squares
    i. The positions of selected crystals in our example are circled in orange in Figure 2.
  c. Bring each selected crystal to the eucentric tilting position (Figure 3)

17. Cool down the dual beam transfer station to liquid nitrogen temperatures
18. Transfer the vitrified TEM grids to the dual beam transfer station
  a. If these grids will be loaded into an autoloader equipped TEM, assure the TEM grids are properly prepared with the appropriate autogrid clips.
    i. Customized autogrid clips have been designed for using the FIB/SEM to make lamellae of biological materials. These clips have a thinner edge on one side to allow for a shallower milling angle. This is advantageous for tomography experiments. For MicroED experiments, milling at higher angles is not necessarily a detriment, as this exposes the thinnest face to the TEM beam at a variable angle, and can be used to optimize data collection. We find that either clips work well for preparing crystal lamellae.
19. Transfer the grids into the dual beam shuttle for transfer into the FIB/SEM dual beam instrument (Figure 1)
  a. Take special care to note and load the grids in the proper orientation – the carbon side of the grid where the crystals are should be facing outward, and the clip ring (if present) should be oriented toward the shallower side of the ring or the appropriate ring orientation identifier (Figure 1).
    i. In our lab, we often mark an edge of the clip ring with a dot using a marker to identify the grid orientation.
20. Transfer the grids inside of the dual beam sample shuttle into the dual beam instrument using the pumped down transfer rod.
21. Allow the dual beam instrument to come back to optimal vacuum state after loading
22. Use the sputter coating system to apply a layer of platinum to the grids prior to further inspection
  a. It is often advisable to use two sets of coatings, a fine coating and then rough coating. The typical settings are a fine sputter coating at 1 kV 7 mA for 30 s, and a rough sputter coating at 1 kV 30 mA for 30 s. This typically deposits 100–300 nm of platinum on the loaded samples.
    i. The system used in this protocol uses nitrogen gas to cool the stage. The sample may heat up during the sputter coating process, and may lead to severe ice contamination of the protein crystals or crystallization of the vitreous ice inside of the protein crystals. This problem can be alleviated by increasing the nitrogen gas flow during sputter coating. Ice contamination has not been observed since this adjustment to the crystal milling protocol was adopted.
  b. A carbon rich platinum layer can also be deposited using the gas injection system to either further supplement the sputter coated platinum or completely replace it. This layer is often very thick. The amount of time needed to mill a lamella will increase with thicker layers of platinum.
23. Screen the loaded grids for appropriate crystals for ion-beam milling
  a. Take an all-grid map or single image of the entire grid to judge the quality of the sample preparation steps. The grid should have visible grid bars, holes in the holey carbon, and have crystals on the carbon film with vitrified liquid still surrounding them (Figure 2)
    i. It is advisable to use commercial software, such as MAPS (Thermo-Fisher) or the microscope’s positioning system to store positions on the stage where potential crystals are visible.

**Figure 3:**
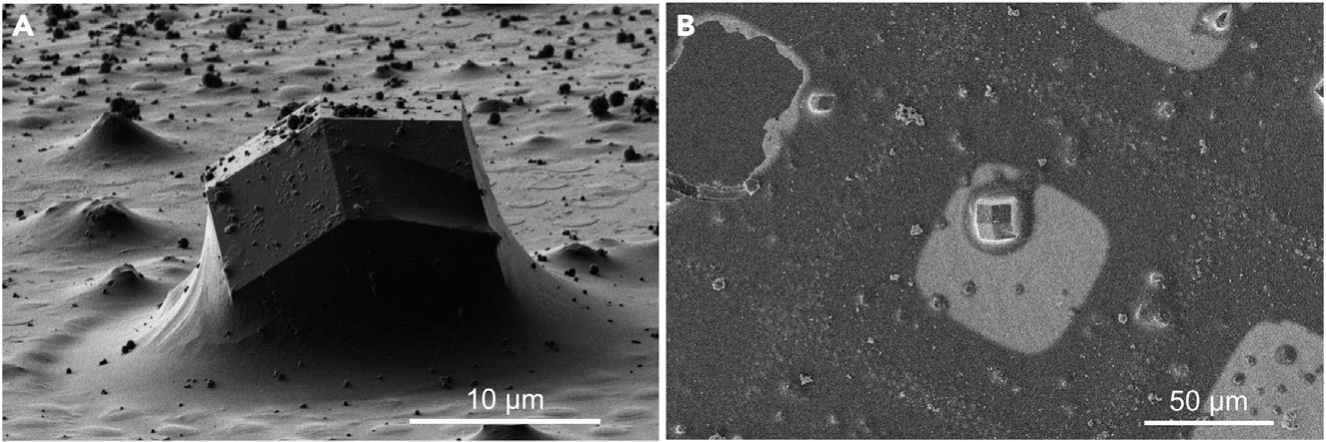
Crystal identification and alignment Views of a selected crystal in the ion-beam (A) and electron beam (B) after successfully bringing the sample to the eucentric position, where the beams are approximately coincident.
  d. Go to a selected crystal using the dual beam user interface and tilt to the milling position
  e. Take a high-resolution image in both SEM and ion-beam to assure the beams are aligned to the same point at the specified milling angle (Figures 3A and 3B)
24. Mill selected crystals into thin lamellae
  a. Rough mill away the bulk material from above and below the selected crystal using rectangular patterns (Figure 4B, blue squares)
    i. Rough milling can be safely conducted using rectangular milling patterns at beam currents between 100 pA–500 pA. The rectangular milling patterns are essentially areas that are constantly imaged and allow the user to directly observe the sample as it is destroyed by ion beam. It is advisable to time how long it takes for the beam to clear away this area and note how this correlates to the calculated time suggested by the software to mill away this thickness of pure silicon.
    ii. All ion beam milling steps in this protocol are conducted using a beam of gallium ions with an accelerating voltage of 30 kV.

**Figure 4:**
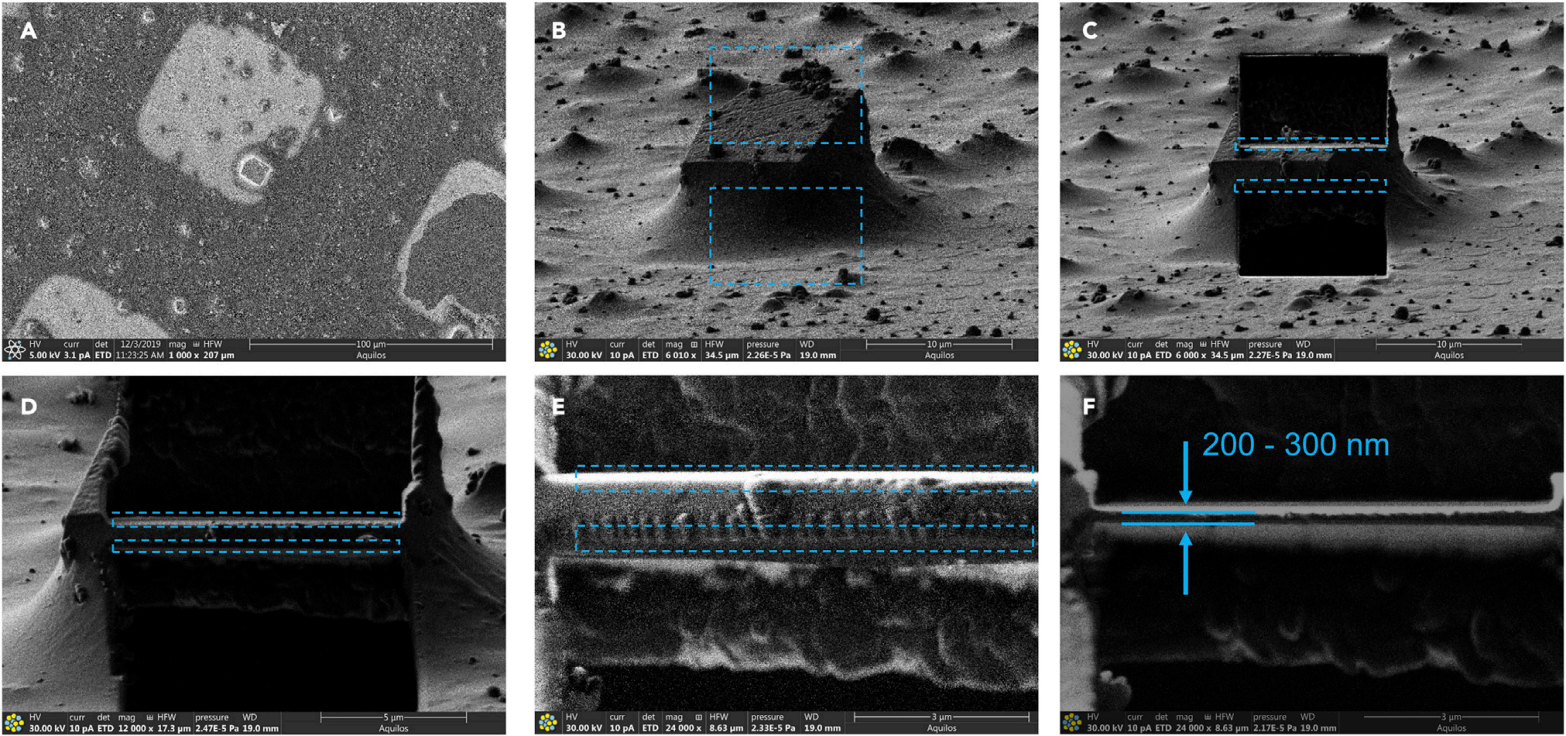
The milling process by the focused ion-beam The crystal prior to milling as seen in the electron (A) and ion-beam (B). The crystal after rough milling (C), fine milling (D), finer milling (E), and polishing (F). The light blue boxes indicate where milling was conducted in order to achieve the following frame. The approximate final thickness is indicated in (F).
  b. Begin the fine milling process to slowly thin the rough lamella into a sharp edged slab. This is done in small steps in order to monitor the thickness and assure the specimen is not damaged due to drift of the beams (Figures 4C and 4D).
    i. Fine milling is also conducted using rectangular milling patterns at ion beam currents of 30–100 pA.
  c. Polish the lamella to the proper thickness and assure the lamella is perfectly flat (figure 4E).
    i. Polishing is conducted at ion beam currents of 1.5–30 pA, and almost always uses a cleaning cross section pattern. This pattern spends all of the calculated time at each position in the rectangular pattern at once and rasters through each line sequentially. This often results in much sharper, flatter lamellae, but removes the live feedback obtained by milling with the rectangular cross sections. Experimenting with what works best for each sample may be necessary.
  d. Take a final image of the lamella using the ion-beam at a high magnification, a very low beam current, and measure the final thickness of the polished lamella (Figure 4F).
    i. Lamella are typically polished at a current of 10–30 pA by gallium ions accelerated at 30 kV.
    ii. It is possible to monitor the milling progress using the scanning electron beam (Figure 5). This allows to also monitor whether the milling has successfully penetrated through whatever material might be below the crystal on the grid. Empty, or completely cleared areas, appear as dark, featureless bands in front of and behind the lamella. It is possible that imaging the lamella in the SEM may damage the crystalline order and reduce final resolution. However, this has not been investigated to date.

**Figure 5:**
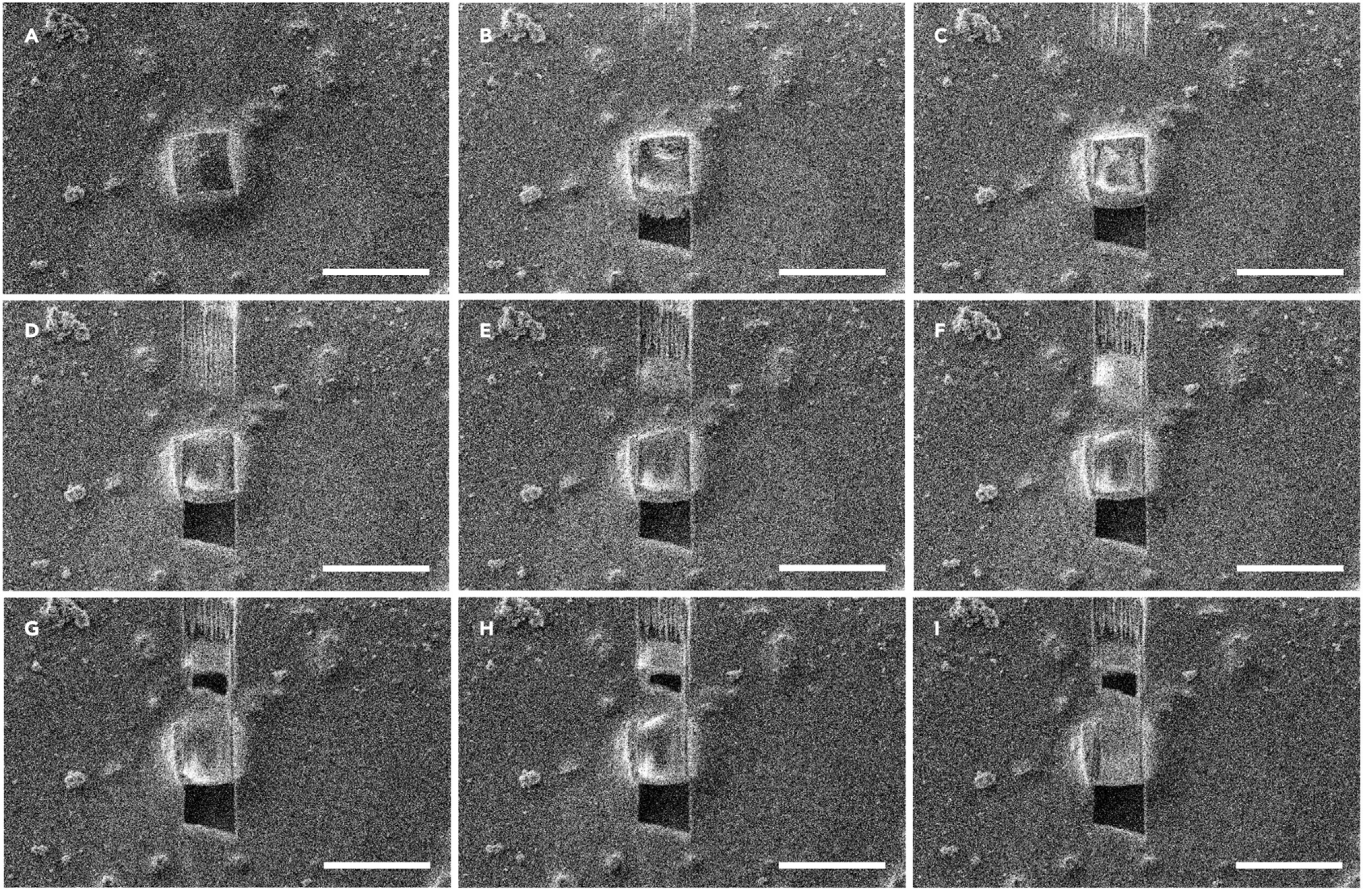
Monitoring the milling process using the SEM Starting from the unmilled crystal in (A), the milling process is monitored by taking a single exposure in using the electron beam until the process is finished (B–I). All scale bars 25 μm.
  e. Repeat steps (a) – (d) for all selected crystals
    i. It is advisable to limit the total number of crystals milled in a single session to perhaps 1–20. Current dual-beam systems have relatively poor vacuum and cooling systems when compared to modern TEMs. Over time, ice builds up over the sample in addition to chunks of milled material finding its way back on the grid and appearing as speckled muck or dirt. For example, the system used in this study was benchmarked at 30–50 nm of ice buildup per hour. Using approximately 30 min per lamella, we could expect the 10^th^ lamella to have nearly zero ice contamination, but the first lamellae could be 3–5 x thicker than anticipated. It is possible to reduce the issue of contamination by conducting the milling steps in series for each lamella, rather than by finishing a lamella complete prior to moving to the next. For example, all 10 could be bulk milled, then fine milled, etc. This process helps, but only marginally improve outcomes. It requires much more effort, and the polishing steps typically take at least 1/3^rd^ of the total milling time per lamella.
25. Transfer the grids with milled lamellae into a liquid nitrogen storage dewar until the TEM is available for use.
  a. It is advisable to load the grids directly into a cryo-cooled TEM without storing them for large periods of time in a storage dewar. One large ice crystal getting stuck to a lamella could ruin hours of work. Plan your experiments accordingly.

**Pause point:** 5 min–1 week

### Collection of MicroED data

**Timing: 0.5–12 h**

**Figure 6:**
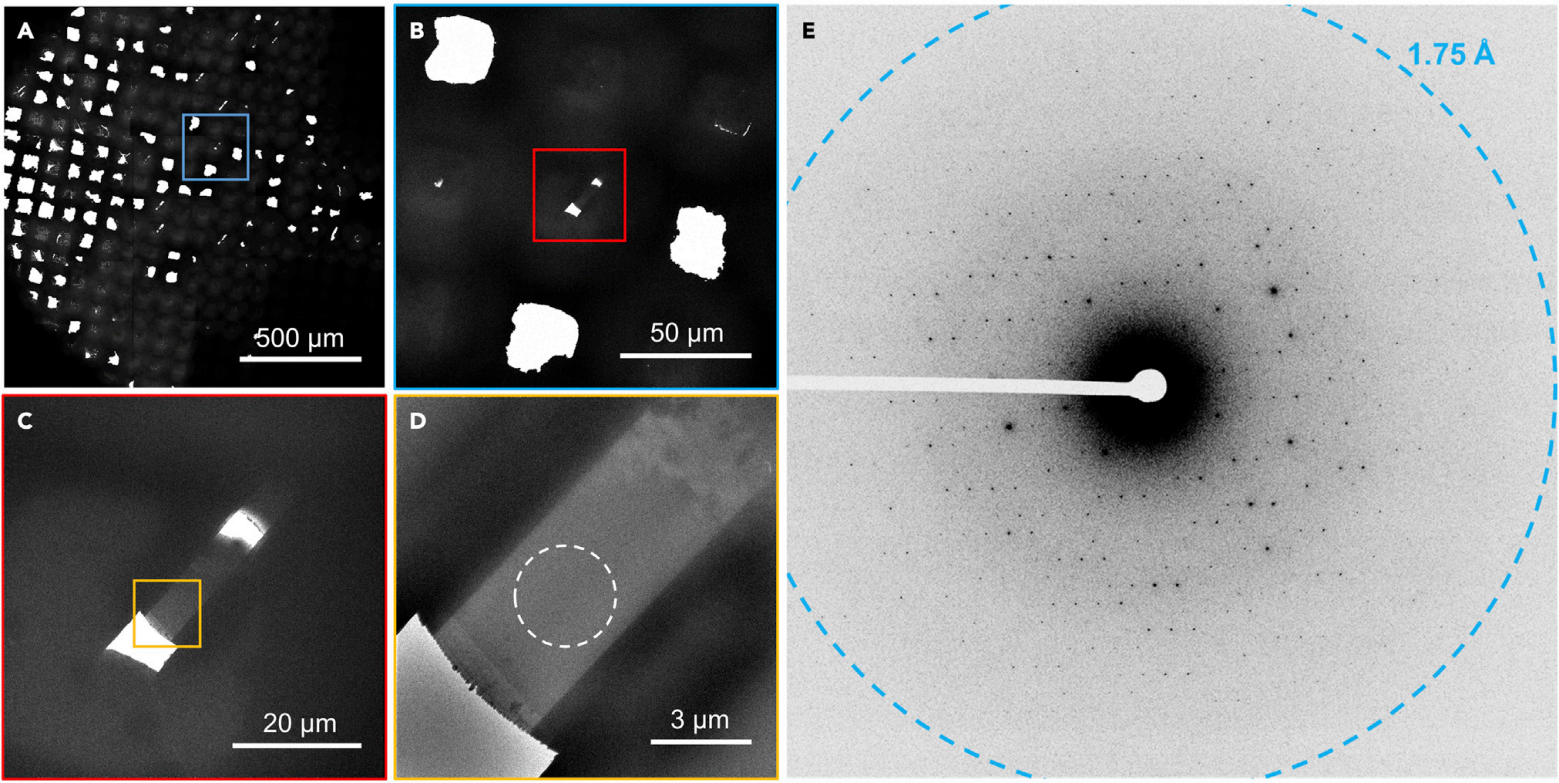
MicroED data collected on a cryogenically cooled TEM (A) The all-grid atlas used to identify milled lamellae. (B–D) Higher magnification images of an identified lamella on the grid. (E) MicroED data from the lamella. Each frame is highlighted and corresponds to the colored region in the frame prior. The white, dashed circle indicates the size and position of the selected area aperture used to collect the MicroED data.

Here, explicit details for the collection of continuous rotation MicroED data are described. These steps assume that the TEM is well aligned for collecting both imaging and diffraction data (Figure 6).

26. Remove the grids containing milled lamellae from the storage dewar and load them into a cryogenically cooled TEM.
  a. Carefully load the grid into either the side entry holder or autoloading system such that the alpha tilt of the stage travels perpendicular to the milling direction (Figure 1). This is absolutely critical to collecting an optimal wedge of MicroED data.
27. Screen the loaded grids for milled lamella
  a. Take an all-grid atlas/montage of each sample
  b. Identify milled crystals by the clear stipe of missing material (Figure 6)
  c. Add the coordinates of each lamella to a list for further investigation

***Note:*** We find that approximately 90% of the lamellae that are successfully milled will survive if loaded directly from the FIB/SEM into the TEM. However, the survival rate drops to approximately 50% if stored for > 1 week in a storage dewar or transported between physical sites.

28. Prepared the identified lamellae for data collection
  a. For each site, take a high magnification image (Figure 6)
  b. If the lamella is not contaminated or broken, bring each site to the eucentric position
29. Collect continuous rotation MicroED data from each lamella(de la Cruz et al., 2019; Martynowycz et al., 2019a; Shi et al., 2016)
30. Remove the grids and either store them for future use or discard them as needed

***Note:*** It is unlikely that the milled lamellae will survive or be useful after milling and loading into the TEM. However, we find that grids with many crystals can be useful for additional milling at a later date. Here, contamination can build up on top of the platinum layers, but the underlying crystals are still preserved. Any buildup of contamination after platinum coating can easily be removed by milling later.

**Pause point:** one day

### MicroED data processing

**Timing: 0.5 h–12 h**

The steps of converting continuous rotation MicroED data into crystallographic format, reducing the data, and determining the structure by molecular replacement are given in detail. There are many methods and approaches to processing diffraction data. A simple method using the command line on either a Mac or Linux based system is described.

31. Convert the continuous rotation MicroED movies into a standard crystallographic format for further processing using the freely available MicroED-tools (cryoem.ucla.edu) (Hattne et al., 2015).
  a. Frames are often collected as TIF, MRC, SER, or many additional formats by the TEM detector software.
  b. In this case, the data were collected as single MRC files containing a continuous sweep of data from one crystal.
  c. Converting from MRC to SMV requires experimental parameters that will be placed into each image header. These headers are read by processing software and are critical to correctly processing the data.
32. Index, integrate, scale, and merge the MicroED data
  a. Each step may be done using standard crystallographic software, such as XDS (Kabsch, 2010a) for initial indexing and integration, and then programs such as POINTLESS (Evans and Murshudov, 2013) to determine the crystallographic space group, AIMLESS (Evans and Murshudov, 2013) for scaling and merging. Several downstream refinement programs require merged amplitudes rather than intensities, such as REFMAC5 (Kovalevskiy et al., 2018), and these intensities can be converted to amplitudes using CTRUNCATE (Winn et al., 2011).
  b. In this report, all seven crystals were merged together using XSCALE (Kabsch, 2010b). The relevant statistics may be found in Table 1.

**Table 1:** MicroED structure of lysozyme from milled lamellae

Milled lysozyme	
Wavelength (Å)	0.0251
Resolution range (Å)	34.18–1.75 (1.81–1.75)
Space group	P 43 21 2
Unit cell (a,b,c)(Å) (a,b,g)(°)	77.33 77.33 38.11 90 90 90
Total reflections (#)	305349 (20448)
Unique reflections (#)	12144 (1177)
Multiplicity	25.1 (17.4)
Completeness (%)	99.98 (100.00)
Mean I/sigma(I)	10.23 (1.38)
Wilson B-factor	24.52
R-merge (%)	0.2314 (1.805)
R-meas (%)	0.2363 (1.86)
R-pim	0.047 (0.4368)
CC_1/2_	0.994 (0.578)
CC∗	0.998 (0.856)
Reflections used in refinement	12144 (1177)
Reflections used for R-free	561 (62)
R-work (%)	17.51 (29.40)
R-free (%)	22.27 (31.91)
Number of non-hydrogen atoms (#)	1089
macromolecules (#)	1001
ligands (#)	3
solvent (#)	85
Protein residues (#)	129
RMS (bonds)	0.006
RMS (angles)	0.79
Ramachandran favored (%)	96.85
Ramachandran allowed (%)	3.15
Ramachandran outliers (%)	0
Rotamer outliers (%)	0.95
Clashscore	3.57
Average B-factor (Å^2^)	25.7
Macromolecules	25.51
Ligands	32.52
Solvent	27.71
33. Determine the structure using molecular replacement
  a. This structure was determined using PHASER (McCoy et al., 2007) using PDB 5K7O as a search model (de la Cruz et al., 2017).
    i. The search was conducted using electron scattering factors. The search procedure looks for solutions in all possible space groups, since the integration program was only confident enough to determine the point group P422, but not determine the potential screw axis. The solved structure was placed in space group P4_3_2_1_2 (#96).
    ii. It is good practice to copy the R free set from the molecular replacement model to the new dataset – extending to the new resolution if needed – in order to remove additional model bias. If this is a new structure, add a new set of R free flags (Winn et al., 2011).
34. Refine the protein structure determined by MicroED
  a. Open the molecular replacement model and map to inspect the solution.
    i. This is typically done in a program such as COOT (Emsley and Cowtan, 2004)
  b. Refine the structure using the original file of merged reflections and R free flags using electron scattering factors.
    i. A typical refinement will refine the individual B factors and atomic positions. This lysozyme structure was refined using PHENIX (Adams et al., 2010).
  c. Add ordered solvent and ions to the structure as they appear in the density
  d. Repeat manual inspection of the maps between rounds of structure refinement until satisfied with the model. The final map should accurately describe the location of all the residues in the protein (Figure 7).

**Figure 7:**
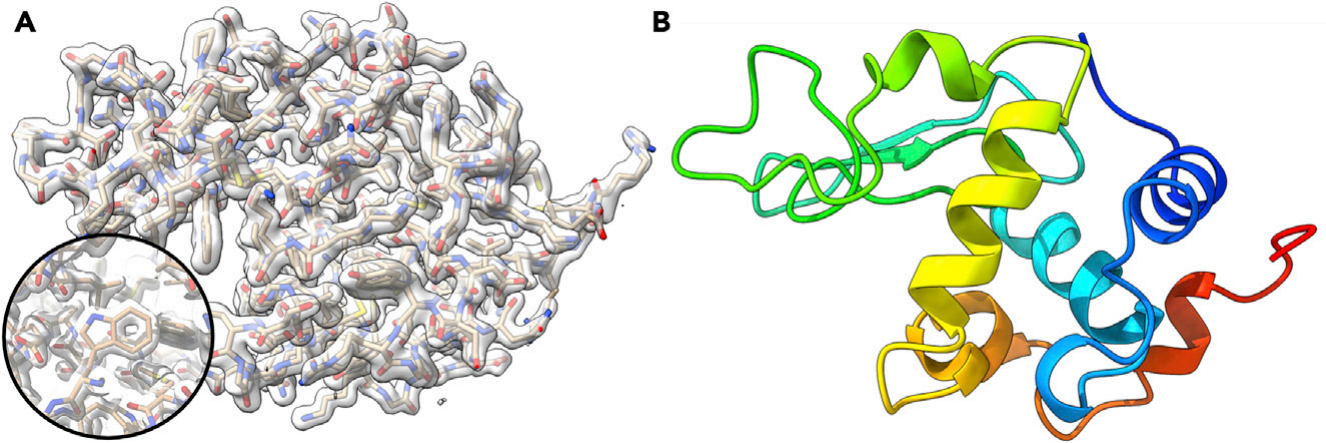
The structure of lysozyme from milled lamellae (A and B) (A) The 2F_o_ – F_c_ map contoured at the 1.5 σ level, and (B) the final structure of lysozyme determined by MicroED from seven merged crystalline lamellae.

## Expected outcomes

It is expected that between 5 and 15 thin lamellae of protein crystal will be prepared after one day of ion-beam milling experiments. These lamellae are between 100 – 300 nm thick and 0.5–10 μm wide with similar depths (Figures 4 and 5). MicroED data are collected from each lamella, and the data are integrated and merged as needed. A high quality structure is determined (for example by molecular replacement) and refined using electron scattering factors.

## Limitations

There are important limitations to milling protein crystals for MicroED experiments. The largest hurdle is generating TEM grids with identifiable protein crystals on them. Blotting grids with protein crystals can be sensitive to the conditions of the blotting chamber, such as the relative humidity, temperature, blotting time, blotting force, and the method of blotting (back blotting, front blotting, etc.)(Martynowycz et al., 2020a, 2020b). The protocol we present is the typical approach used in the Gonen lab, and works in most cases. However, each sample may behave differently depending on the protein and the crystallization conditions. The standard grids used for milling crystals have been 200 mesh holey carbon films with 2 μm holes (Cu 200 R 2/2) that are blotted gently from the back. In principle, any grid could be used for milling. However, this hole shape and spacing has typically been the most successful of the standard holey carbon films. There have been no experimental results suggesting that carbon films are any better or worse than gold foils or lacey carbon films. This may be an interesting avenue to explore. It is noted that wider grid meshes such as 200 are greatly preferred to 300 or 400 meshes. These grids have more area amenable to milling, but are more prone to breaking during transfer steps. The grids generated for this protocol were blotted using a Leica GP2 EM plunger that blots from one side, and was used to blot the grids from the back. Other vitrification robots such as the Thermo Scientific Vitrobot can also be used to prepare grids for milling. In our lab, blotting from both sides of the grid usually results in the majority of the crystals lost. Grids have also been successfully generated using a manual plunger inside of a cold room where all the blotting is conducted without an environmentally controlled sample chamber.

After suitably preparing grids, the next hurdle involve adequately protecting the crystals from the electron and ion beams during the machining process (Zhou et al., 2019). Many crystals can withstand both beams for prolonged periods without any adverse effects on the ultimate resolution of the data, whereas others appear to lose all crystalline order after just a few exposures from the ion beam – even at only 1–10 pA of current (Martynowycz et al., 2019b; Zhou et al., 2019). Here, we used a sputter coated platinum layer that will protect most crystals from the beams long enough to prepare good lamellae. It is possible that additional layers of thicker, carbon rich platinum or other materials from a gas injection system (GIS) may improve the quality of data for more sensitive specimens (Beale et al., 2020). The range of data collection by MicroED is limited by the rotation range on the TEM stage - typically −70° to +70°. In practice, collection of MicroED data from each lamella are not limited by the physical range of the stage, but rather by radiation damage or the grid bar coming into view. We have previously detailed the effects of radiation damage on protein crystals, and suggest that a maximum exposure of approximately 3 e^-^ Å^−2^ for data to 3 Å as a soft limit (Hattne et al., 2018). The range of data this corresponds to depends strongly on the sensitivity of the detector used and the quality of the diffraction generated by the crystalline lamellae. For example, a typical exposure rate of 0.01 e^-^ Å^−2^ s^−1^ would allow for 300 s of data per lysozyme lamellae. As such, covering the entire rotation range of 140° in 300 s would require a rotation speed of 0.466° s^−1^. To collect this wedge using a more conservative exposure of 1 e^-^ Å^−2^ s^−1^ would require rotation at 1.4°s^−1^. These parameters need to be optimized by the user based on the crystal symmetry and geometry and crystal susceptibility to succumb to radiation damage.

Throughput of MicroED data collection from milled lamellae is limited milling time. Current dual beam systems have rather poor vacuum systems compared to modern TEMs. As such, the samples inside of the FIB/SEM continually build up ice over time that contaminates the milled lamellae. We estimate this to be approximately 25–50 nm of ice per hour of milling operation. If an individual lamellae 200 nm thick can be prepared by a skilled operator in approximately 30 min after alignment and setup, then we can expect the sample to significantly degrade in quality after only preparing an additional 2–3 lamellae sequentially. We alleviate this issue slightly by completing each general milling task for each crystal- e.g., trenching, rough milling, fine milling, polishing – rather than completely preparing each lamellae before proceeding to the next site. The poor vacuum and very large stages in modern cryo dual beam systems result in substantial drift and inaccuracies in recalling exact stage positions. Though there has been recent success in scripting or attempting to automate the milling process for cellular lamellae (Buckley et al., 2020; Zachs et al., 2020), no such pipelines have been demonstrated for protein crystals. Protein crystals tend to be quite fragile since they typically contain significant amounts of solvent and solvent channels. This makes automation more challenging but clearly would benefit to increase throughput.

## Troubleshooting

### Problem 1

The grids appear entirely covered in thick ice after loading into the dual-beam system.

### Potential solution

This is likely caused by either inefficient blotting or the solvent being too viscous to blot by standard back-blotting approaches. The grids can be blotted for longer periods of time, or the blotting strategy can be changed to blot from the front or both the front and the back. It is also possible to rinse the grid with a few uL for mother liquor after the first blot, and blot again in order to rinse away any buildup of material. More viscous materials, such as lipid bicelles or LCP may require thinning or converting the phase using a second solution of PEG to allow the lipids matrix to flow.

### Problem 2

There are no visible crystals on the grid.

### Potential solution

This can happen for a variety of reasons ranging from the crystals being destroyed by pipetting them on the grid to the blotting process itself is destroying the crystals. Blotting using a vitrification robot is advantageous as the chamber humidity and temperature can be controlled. Blotting at 4°C and 95% humidity often prevents the crystals from dehydrating during the blotting process. Crystals may also be flat sheets that are very difficult to detect. In these cases, SEM imaging may help locate crystals (Martynowycz et al., 2020a).

### Problem 3

The crystals are being destroyed by the ion-beam.

### Potential solution

Increase the amount of platinum coating on the sample. For the most sensitive crystals, adding a much thicker (0.2–5 μm) of carbon-rich platinum using the gas injection system may be necessary. The beam may and/or the stage can drift or shutter during the milling process, and this will very quickly destroy a lamella, especially in the polishing stages. Both the ion and electron beams should be carefully calibrated and aligned frequently to reduce drift. Limiting the milling times for individual cross sections to only a few minutes at a time with intermediate checks for movement/drift are often advantageous.

### Problem 4

The crystals are getting covered by ice before MicroED data can be collected.

### Potential solution

Be sure that the storage dewar is regularly cleaned and minimize the time between milling and collecting MicroED data. Transferring grids between the FIB/SEM and the TEM should also be done carefully, with the liquid nitrogen being filtered when cooling down the tools and equipment. Always thaw and dry your tools prior to using them to manipulate grids at cryogenic conditions. Do not forget to allow your tools to equilibrate/boil in the liquid nitrogen prior to touching anything near your samples – the transfer of heat can destroy the specimen.

### Problem 5

The sides of the crystal come into view during rotation, preventing the collection of MicroED data at higher tilts.

### Potential solution

The rotation axis of the TEM should be known prior to collecting MicroED data. The lamellae should rotate such that the milling direction is perpendicular to the rotation axis. This will assure the maximum range of rotation when collecting data. If there is some external factor making this impossible, it is possible to clear the entire top of the crystal when rough milling rather than creating a trench through the crystal, though this will only marginally improve the range of measurement.

### Problem 6

Few crystals survive between selection and final MicroED data collection.

### Potential solution

Currently, milling protein crystals is a manual and labor intensive process. The loss of any lamellae constitutes a large loss of time and effort. Experience has shown that the greater the time between finishing a lamella and collecting data in the TEM, the higher the chance a lamella will break, bend, or get covered in contamination. For example, early crystal milling experiments were conducted days prior to data collection and involved transportation in a storage dewar between Pasadena, CA and Los Angeles, CA – a 30-mile trip taking anywhere between 20 and 120 min. These experiments saw lamella survival rates as low as 30%. However, current practices have the grid transferred directly from the FIB/SEM to the TEM in the same room. This transfer can be done even when data will not be collected for several days if the TEM is equipped with a reliable auto-loader. In these cases, the lamella survival rate has been as high as 100%, and is typically at least 80%. With careful manipulation during the transfer steps and ice-free equipment, most lamella should survive to data collection.

## Resource availability

### Lead contact

Further information and requests for resources and reagents should be directed to and will be fulfilled by the lead contact, Tamir Gonen (tgonen@g.ucla.edu).

### Materials availability

All materials used in this protocol are commercially available.

### Data and code availability

The coordinates, structure factors, and EM maps have all been deposited in the PDB under accession codes PDB 7MRB and EMD-23957.
